# Supplementary material for: Preconception Folic Acid Supplement Use in Immigrant Women (1999–2016)
Source: Nutrients. 2019 Sep 27;11(10):2300. doi: 10.3390/nu11102300 (PMC6836227; doi:10.3390/nu11102300)
Supplement: Supplementary file 1 [file nutrients-11-02300-s001.zip › Nilsen_Suppl_Figure_1.pdf]

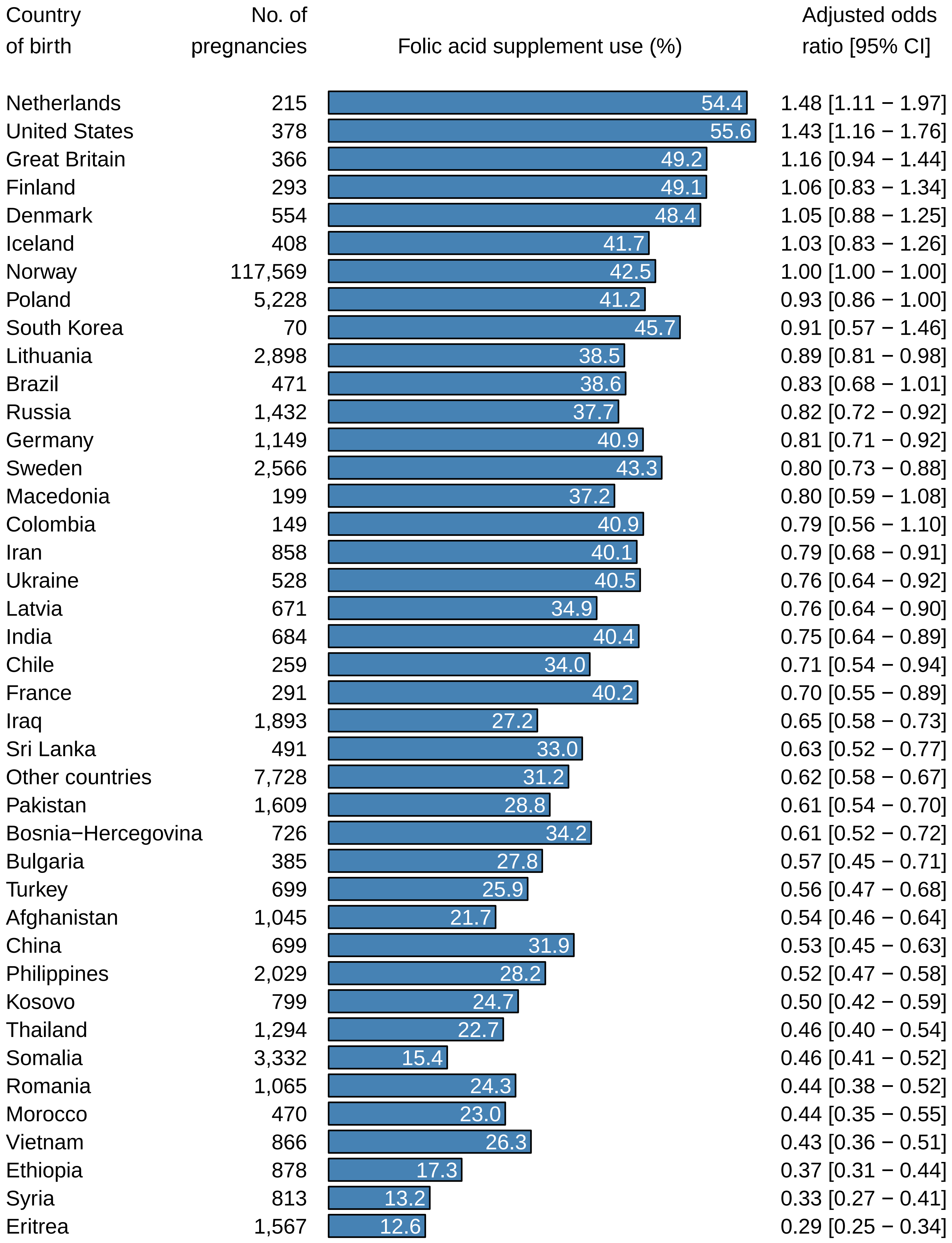

Country of birth
No. of pregnancies
Folic acid supplement use (%)
Adjusted odds ratio [95% CI]
Netherlands 215 54.4 1.48 [1.11 − 1.97]
United States 378 55.6 1.43 [1.16 − 1.76]
Great Britain 366 49.2 1.16 [0.94 − 1.44]
Finland 293 49.1 1.06 [0.83 − 1.34]
Denmark 554 48.4 1.05 [0.88 − 1.25]
Iceland 408 41.7 1.03 [0.83 − 1.26]
Norway 117,569 42.5 1.00 [1.00 − 1.00]
Poland 5,228 41.2 0.93 [0.86 − 1.00]
South Korea 70 45.7 0.91 [0.57 − 1.46]
Lithuania 2,898 38.5 0.89 [0.81 − 0.98]
Brazil 471 38.6 0.83 [0.68 − 1.01]
Russia 1,432 37.7 0.82 [0.72 − 0.92]
Germany 1,149 40.9 0.81 [0.71 − 0.92]
Sweden 2,566 43.3 0.80 [0.73 − 0.88]
Macedonia 199 37.2 0.80 [0.59 − 1.08]
Colombia 149 40.9 0.79 [0.56 − 1.10]
Iran 858 40.1 0.79 [0.68 − 0.91]
Ukraine 528 40.5 0.76 [0.64 − 0.92]
Latvia 671 34.9 0.76 [0.64 − 0.90]
India 684 40.4 0.75 [0.64 − 0.89]
Chile 259 34.0 0.71 [0.54 − 0.94]
France 291 40.2 0.70 [0.55 − 0.89]
Iraq 1,893 27.2 0.65 [0.58 − 0.73]
Sri Lanka 491 33.0 0.63 [0.52 − 0.77]
Other countries 7,728 31.2 0.62 [0.58 − 0.67]
Pakistan 1,609 28.8 0.61 [0.54 − 0.70]
Bosnia−Hercegovina 726 34.2 0.61 [0.52 − 0.72]
Bulgaria 385 27.8 0.57 [0.45 − 0.71]
Turkey 699 25.9 0.56 [0.47 − 0.68]
Afghanistan 1,045 21.7 0.54 [0.46 − 0.64]
China 699 31.9 0.53 [0.45 − 0.63]
Philippines 2,029 28.2 0.52 [0.47 − 0.58]
Kosovo 799 24.7 0.50 [0.42 − 0.59]
Thailand 1,294 22.7 0.46 [0.40 − 0.54]
Somalia 3,332 15.4 0.46 [0.41 − 0.52]
Romania 1,065 24.3 0.44 [0.38 − 0.52]
Morocco 470 23.0 0.44 [0.35 − 0.55]
Vietnam 866 26.3 0.43 [0.36 − 0.51]
Ethiopia 878 17.3 0.37 [0.31 − 0.44]
Syria 813 13.2 0.33 [0.27 − 0.41]
Eritrea 1,567 12.6 0.29 [0.25 − 0.34]
