# Supplementary material for: Preconception Folic Acid Supplement Use in Immigrant Women (1999–2016)
Source: Nutrients. 2019 Sep 27;11(10):2300. doi: 10.3390/nu11102300 (PMC6836227; doi:10.3390/nu11102300)
Supplement: Supplementary file 1 [file nutrients-11-02300-s001.zip › Nilsen_Suppl_Table_1.pdf]

Table S1. Percentages and odds ratios for preconception folic acid supplement use by maternal immigration category and period of birth.

Immigration category	Period of birth					
	1999-2001	2002-2004	2005-2007	2008-2010	2011-2013	2014-2016
Non-immigrant women[a]						
No. of pregnancies	144,416	134,892	135,104	136,662	125,351	117,569
Folic acid supplement use, No. (%)	17,059 (11.8)	25,353 (18.8)	41,736 (30.9)	51,291 (37.5)	46,394 (37.0)	49,982 (42.5)
Norwegian–born women with one foreign–born parent						
No. of pregnancies	5,389	5,322	5,616	5,922	6,028	6,299
Folic acid supplement use, No. (%)	728 (13.5)	1,065 (20.0)	1,808 (32.0)	2,184 (36.9)	2,171 (36.0)	2,693 (42.8)
Crude odds ratio [95% CI]	1.17 [1.07 - 1.27]	1.08 [1.01 - 1.16]	1.06 [1.00 - 1.13]	0.97 [0.92 - 1.03]	0.96 [0.91 - 1.01]	1.01 [0.96 - 1.06]
Adjusted odds ratio [95% CI][b]	1.09 [1.00 - 1.18]	1.01 [0.94 - 1.08]	1.00 [0.94 - 1.07]	0.95 [0.90 - 1.01]	0.97 [0.91 - 1.02]	0.96 [0.91 - 1.02]
Foreign–born women with two Norwegian–born parents						
No. of pregnancies	1,726	1,727	1,810	1,820	1,648	1,480
Folic acid supplement use, No. (%)	219 (12.7)	357 (20.7)	563 (31.1)	676 (37.1)	581 (35.3)	600 (40.5)
Crude odds ratio [95% CI]	1.08 [0.94 - 1.25]	1.13 [1.00 - 1.16]	1.01 [0.91 - 1.12]	0.98 [0.89 - 1.08]	0.93 [0.84 - 1.03]	0.92 [0.83 - 1.03]
Adjusted odds ratio [95% CI] [b]	0.95 [0.82 - 1.11]	1.00 [0.88 - 1.13]	0.93 [0.84 - 1.04]	0.93 [0.84 - 1.02]	0.88 [0.79 - 0.98]	0.87 [0.78 - 0.97]
Foreign–born women with one Norwegian–born parent						
No. of pregnancies	1,198	1,150	1,182	1,259	1,064	1,053
Folic acid supplement use, No. (%)	165 (13.8)	261 (22.7)	388 (32.8)	464 (36.9)	377 (35.4)	460 (43.7)
Crude odds ratio [95% CI]	1.19 [1.01 - 1.41]	1.27 [1.10 - 1.46]	1.09 [0.97 - 1.24]	0.97 [0.86 - 1.09]	0.93 [0.82 - 1.06]	1.05 [0.93 - 1.19]
Adjusted odds ratio [95% CI] [b]	1.11 [0.93 - 1.31]	1.20 [1.03 - 1.39]	1.02 [0.89 - 1.16]	0.91 [0.81 - 1.03]	0.91 [0.80 - 1.04]	1 [0.88 - 1.14]
2nd generation immigrant women [c]						
No. of pregnancies	575	777	1,070	1,473	1,817	2,253
Folic acid supplement use, No. (%)	41 (7.1)	91 (11.7)	222 (20.8)	353 (24.0)	462 (25.4)	860 (38.2)
Crude odds ratio [95% CI]	0.57 [0.42 - 0.79]	0.57 [0.46 - 0.71]	0.59 [0.50 - 0.68]	0.52 [0.46 - 0.59]	0.58 [0.52 - 0.65]	0.83 [0.77 - 0.91]
Adjusted odds ratio [95% CI] [b]	0.69 [0.50 - 0.94]	0.66 [0.53 - 0.83]	0.60 [0.51 - 0.70]	0.54 [0.47 - 0.61]	0.60 [0.54 - 0.67]	0.77 [0.71 - 0.85]
1st generation immigrant women [d]						
No. of pregnancies	20,711	24,506	28,741	36,651	43,570	48,055
Folic acid supplement use, No. (%)	1,578 (7.6)	2,527 (10.3)	5,095 (17.7)	8,026 (21.9)	10,301 (23.6)	15,265 (31.8)

Crude odds ratio [95% CI]	0.62 [0.58 - 0.65]	0.50 [0.48 - 0.52]	0.48 [0.47 - 0.50]	0.47 [0.45 - 0.48]	0.53 [0.51 - 0.54]	0.63 [0.62 - 0.64]
Adjusted odds ratio [95% CI] [b]	0.65 [0.61 - 0.69]	0.54 [0.52 - 0.57]	0.51 [0.49 - 0.53]	0.50 [0.48 - 0.51]	0.56 [0.54 - 0.57]	0.68 [0.66 - 0.70]

Abbreviations: CI, confidence interval. [a] Reference group: Norwegian-born women with two Norwegian-born parents (non-immigrants). [b] Adjusted for year of birth, maternal age, marital status, parity, geographical region, education and income. [c] Norwegian-born women with two foreign-born parents. [d] Foreign-born women with two foreign-born parents.
